# Administration of mircoRNA‐135b‐reinforced exosomes derived from MSCs ameliorates glucocorticoid‐induced osteonecrosis of femoral head (ONFH) in rats

**DOI:** 10.1111/jcmm.16006

**Published:** 2020-10-22

**Authors:** Xiang Zhang, Jiong‐ming You, Xiao‐jun Dong, Yang Wu

**Affiliations:** ^1^ Department of Orthopedics Wenzhou Hospital of Integrated Traditional Chinese and Western Medicine Zhejiang University of Traditional Chinese Medicine Wenzhou China; ^2^ Department of Orthopaedics Wuhan Hospital of Traditional Chinese Medicine Wuhan China; ^3^ Department of Internal Medicine of TCM Wenzhou Hospital of Integrated Traditional Chinese and Western Medicine Zhejiang University of Traditional Chinese Medicine Wenzhou China

**Keywords:** apoptosis, miR‐135b, ONFH, PDCD4, stem cells

## Abstract

Exosomes were found to exert a therapeutic effect in the treatment of osteonecrosis of the femoral head (ONFH), while miR‐135b was shown to play an important role in the development of ONFH. In this study, we investigated the effects of concomitant administration of exosomes and miR‐135b on the treatment of ONFH. A rat mode of ONFH was established. TEM, Western blotting and nanoparticle analysis were used to characterize the exosomes collected from human‐induced pluripotent stem cell–derived mesenchymal stem cells (hiPS‐MSC‐Exos). Micro‐CT was used to observe the trabecular bone structure of the femoral head. Real‐time PCR, Western blot analysis, IHC assay, TUNEL assay, MTT assay and flow cytometry were performed to detect the effect of hiPS‐MSC‐Exos and miR‐135b on cell apoptosis and the expression of PDCD4/caspase‐3/OCN. Moreover, computational analysis and luciferase assay were conducted to identify the regulatory relationship between PDCD4 mRNA and miR‐135b. The hiPS‐MSC‐Exos collected in this study displayed a spheroidal morphology with sizes ranging from 20 to 100 nm and a mean concentration of 1 × 10^12^ particles/mL. During the treatment of ONFH, the administration of hiPS‐MSC‐Exos and miR‐135b alleviated the magnitude of bone loss. Furthermore, the treatment of MG‐63 and U‐2 cells with hiPS‐MSC‐Exos and miR‐135b could promote cell proliferation and inhibit cell apoptosis. Moreover, PDCD4 mRNA was identified as a virtual target gene of miR‐135b. HiPS‐MSC‐Exos exerted positive effects during the treatment of ONFH, and the administration of miR‐135b could reinforce the effect of hiPS‐MSC‐Exos by inhibiting the expression of PDCD4.

## 1 INTRODUCTION

The incidence of osteonecrosis of the femoral head (ONFH) is high in young adults and middle‐aged people. In the United States, the annual incidence of ONFH is ten to twenty thousand of new cases.^1^ The aetiology of ONFH is complex, although all risk factors of ONFH are related to the ischaemia of the femoral head. These ischaemic conditions can lead to the necrosis of osteocytes and the loss of structural integrity in the femoral head.^2^ It has been reported that the apoptosis of osteocytes becomes more obvious in glucocorticoid‐induced ON than that in alcohol‐induced ON. In addition, the apoptosis of osteocytes is absent in ON triggered by sickle cell disease or traumatic events.^3^ It has also been found that the apoptosis of osteocytes and osteoblasts is enhanced in ONFH compared with that in subcapital fracture or regular OA.^4^ As small liposomes with a diameter of <100 nm, exosomes are generated by the invagination of endosomal membrane in multi‐vesicular bodies (MVBs). Subsequently, exosomes are released into the extracellular space via fusion to the plasma membrane.^5^ These extracellular vehicles (EVs) are implicated in the transportation of many essential materials, including proteins, RNAs and cytokines.^1^ In addition, the content in exosomes is protected against degradation by the exosomal membrane. Furthermore, specific ligands located on the surface of exosomes can deliver biochemical materials into target cells by binding to these cells, thus stimulating corresponding biological functions.^6^ In addition, it has been shown that the serum level of exosome is significantly reduced in patients suffering from steroid‐induced ONFH. The ROC analysis has also shown that the level of serum exosome may be used as a diagnostic marker in steroid‐induced ONFH.^7^

As a novel tumour inhibitor, programmed cell death protein 4 (PDCD4) is involved in programmed cell death. Located on chromosome 10q24, the PDCD4 gene plays important roles and its allelic mutations are frequently observed in human cancers. The up‐regulation in PDCD4 expression is seen during apoptosis, indicating that the reduction in PDCD4 expression may render cancer cells anti‐apoptotic.^8^ Furthermore, the expression of miR‐206 is up‐regulated in SANFH specimens along with down‐regulated PDCD4 expression.^9^ Therefore, miR‐206 may promote the development of SANFH by inhibiting the proliferation of osteoblasts and by inducing their apoptosis, which in turn is dependent on the function of PDCD4.^9^

As small and non‐coding RNAs, microRNAs (miRNAs) play essential roles in the post‐transcriptional regulation of gene expression.^10^ By binding to the complementary sequences in the 3′‐untranslated regions (3′‐UTRs) of their target mRNAs, miRNAs induce the translational repression or degradation of their target genes.^11^ It has been shown that miRNAs are present in exosomes during the osteogenic differentiation of bone marrow derived stromal cells (BMSCs). In addition, the expression of miR‐302b, miR‐299‐5p, miR‐219, miR‐203, miR‐135b, miR‐148a, miR‐218, miR‐199b and let‐7a is significantly elevated in exosome samples collected from human BMSCs.^12^ The up‐regulation in miR‐135b was also shown to impair the osteogenic differentiation of BMSCs in patients with multiple myeloma, and it also exhibited protective effect in the development of ONFH via activating the AMPK.^13^, ^14^

It has also been found that the administration of exosomes collected from human‐induced pluripotent stem cell‐derived mesenchymal stem cells (hiPS‐MSC‐Exos) can promote the angiogenesis in ischaemic tissues.^15^ These results suggested that hiPS‐MSC‐Exos may be used as a powerful tool in the treatment of ischaemic diseases, such as ONFH.^16^, ^17^ It was also shown that hiPS‐MSCs‐Exos can prevent bone loss by promoting the angiogenesis in ONFH.^18^

It has been reported that the deregulation of miRNAs is involved in the pathogenesis of ONFH.^19^ In particular, miR‐135b has been shown to play an important role in the development of ONFH.^14^ Furthermore, exosomes were found to exert a therapeutic effect in the treatment of ONFH.^16^, ^17^ In this study, we isolated hiPS‐MSC‐Exos and administered them with miR‐135b in a rat animal model of ONFH, so as to investigate their therapeutic effect in the treatment of ONFH as well as relevant underlying molecular mechanisms.

## 2 MATERIALS AND METHODS

### 2.1 Isolation and characterization of exosomes

Exosomes were isolated from human MSCs using a miRCURY Exosome Isolation Kit (Exiqon) following the kit instructions. In brief, human MSCs were incubated in high‐glucose DMEM containing 10% of foetal bovine serum and 1% penicillin/streptomycin (100 unit/mL streptomycin and 100 μg/mL penicillin). The culture conditions were 37°C, 5% CO_2_ and saturated humidity. After reaching 80% confluency, the SMSCs were rinsed with PBS before being cultured in a FBS‐free MesenGro hMSC medium for 48 hours. Subsequently, the cells were centrifugated respectively at 300 *g* and 2000 *g* (10 minutes each) and the supernatant was pre‐filtered through a 0.22 µm membrane filter (Millipore), ultra‐centrifuged using a 15 mL Amicon Ultra‐15 Centrifugal Filter Unit (Millipore), and finally ultra‐centrifuged for 1 hour at 100 000 *g* in a sterile Ultra‐Clear™ tube (BD). Subsequently, the morphology and distribution of extracted exosomes were measured using Western blotting, transmission electron microscopy (TEM), and dynamic light scattering (DLS), respectively. The expression of markers including CD9, CD63 and CD81 on the surface of the exosomes was measured using Western blotting to confirm the correct identity of these isolated exosomes.

### 2.2 Animal model and grouping

A total of 60 healthy adult female Sprague‐Dawley (SD) rats weighing between 200 and 300 g were purchased from the experimental animal centre of our institute. In order to investigate the therapeutic effect of exosomes and miR‐135b on the treatment of ONFH, a rat model of ONFH was established and the rats were randomly divided into a SHAM group (sham‐operated rats, n = 15), an ONFH group (rats induced of ONFH by GC, n = 15), an ONFH + EXO group (ONFH rats treated with exogenous MSC‐Exos, n = 15), and an ONFH + EXO+miR‐135b group (ONFH rats treated with both exogenous MSC‐Exos and miR‐135b, n = 15). In the ONFH group, methylprednisolone (MPS, Sigma‐Aldrich) at a dose of 20 mg/kg/d was given once daily via intramuscular injection for three weeks to induce ONFH. After the course of injections was finished, the rats were fed with a standard diet and were allowed of free activity for another 3 weeks. In the ONFH + EXO group, ONFH rats were given an administration of 1 × 10^11^ exosomes (100 μL of hiPS‐MSC‐Exos) resuspended in 200 μL of PBS via tail vein injection on a daily basis. In the ONFH + EXO + miR‐135b group, ONFH rats were given daily administration of 1 × 10^11^ exosomes and miR‐135b simultaneously via tail vein injection. All animal experiments were approved by the Institutional Ethics Committee and were conducted in accordance with NIH's Laboratory Animal Care and Use Guide.

### 2.3 Analysis and characterization of bone structure using micro‐computed tomography (micro‐CT)

A high‐resolution micro‐CT system (SkyScan 1272, Bruker micro‐CT) was used to detect the structural characteristics of femoral heads collected from ONFH rats. The collected bone tissues were cut into thin slices and wrapped in a wax film coated with dental wax to prevent drying or bone movement during the scanning process. The voltage of X‐ray was set to 50 kV and the light beam was filtered using a 0.5 mm aluminium filter. Hydroxyapatite models with a density of 250 and 750 mg/cm^3^, respectively, were used to carry out density calibration. The NRecon software (V.1.6.9.8; Bruker micro‐CT) was used for image reconstruction. The trabecular bone was manually isolated from the bone marrow and measured to determine and number of trabeculae, the proportion of bone in tissue samples, trabecular separation, and trabecular thickness. The parameters of the trabecular bone were analysed from the 200th section to the 1400th section on the far‐end of growth plate.

### 2.4 RNA isolation and real‐time PCR

Total RNA content from collected tissue and cell samples was extracted using a TRIzol reagent kit (Invitrogen), and the concentration and purity of RNA were determined via the detection of its optical density value. According to the instructions of a PrimeScript RT reagent Kit (Takara), RNA was reversely transcribed into cDNA. The total volume of RT reaction system is 25 μL and the reaction conditions were set as the following: reverse transcription at 37°C for 3 cycles (15 min each cycle), and inactivation of reverse transcriptase for 5 seconds at 85°C. Real‐time PCR was carried out using an ABI 7900 real‐time PCR system (ABI). The reaction conditions were as follows: pre‐denaturation at 95°C for 4 minutes, and 40 cycles of denaturation at 94°C for 30 seconds, annealing at 58°C for 30 seconds, and extension at 72°C for 1 minute, followed by a final extension cycle at 72°C for 7 minutes. The expression of miR‐135b and PDCD4 mRNA was calculated using the 2^−ΔΔCt^ method^40^ and β‐actin/U6 was used as the internal reference. In this study, the primer sequences used were: miR‐135b‐forward: 5′‐GGCTTTTCATTCCTATGTG‐3′; miR‐135b‐reverse: 5′‐GAACATGTCTGCGTATCTC‐3′; PDCD4‐forward: 5′‐ACTGTGCCAACCAGTCCAAAGG‐3′; PDCD4‐reverse: 5′‐CCTCCACATCATACACCTGTCC‐3′; beta‐actin‐forward: 5′‐CACCATTGGCAATGAGCGGTTC‐3′; beta‐actin‐reverse: 5′‐AGGTCTTTGCGGATGTCCACGT‐3′.

### 2.5 Cell culture and transfection

MG‐63 and U‐2 cells were cultured in DMEM (Gibco, Thermo Fisher Scientific) supplemented with 10% foetal bovine serum. When the cells reached 90% confluence, they were treated with hiPS‐MSC‐Exos or transfected with miR‐135b using Lipofectamine 2000 (Invitrogen) according to the instructions of the manufacturer. At 48 hours post cell treatment, the cells were harvested for subsequent experiments.

### 2.6 Vector construction, mutagenesis and luciferase assay

The 3′ UTR of PDCD4 mRNA containing the binding site of miR‐135b was amplified by PCR and cloned into a pcDNA dual‐luciferase vector (Promega). At the same time, site‐directed mutagenesis was carried out in the miR‐135b binding site to obtain mutant PDCD4, which was also cloned into the pcDNA vector. In the next step, MG‐63 and U‐2 cells were co‐transfected with miR‐135b mimics + wild type/mutant PDCD4 using Lipofectamine 2000. At 48 hours post transfection, the luciferase activity of transfected cells was detected using a dual‐luciferase detection kit (Promega) on a luminometer.

### 2.7 Western blot analysis

Cell and tissue samples were lysed in a RIPA buffer and then centrifuged at 4℃ and 12 000 *g* for 15 minutes to harvest total protein, which was separated by 10% SDS‐PAGE (Bio‐Rad) and transferred onto a polyvinylidene difluoride (PVDF) membrane (Millipore). The PVDF membrane was then blocked in 5% skimmed milk and then incubated at 4°C overnight with primary anti‐PDCD4 and anti‐caspase antibodies (Abcam), followed by incubation with HRP‐labelled secondary antibodies for 60 minutes at room temperature. An enhanced chemiluminescence instrument was utilized to visualize the expression of target proteins.

### 2.8 Apoptosis analysis

The apoptotic profiles of cell and tissue samples were measured using TUNEL and flow cytometry assays, respectively. For flow cytometry assay, an Annexin V‐FITC/PI staining kit (Invitrogen) was used according the kit instructions. For TUNEL assay, a TUNEL kit (Thermo Fisher) was used.

### 2.9 MTT assay

The proliferation of cells was measured using an MTT assay according the kit instructions.

### 2.10 Immunohistochemistry

An immunohistochemistry assay was carried out to observe the expression of PDCD4 in tissue samples. In brief, the collected tissue samples were fixed in 4% formaldehyde, embedded in paraffin, and sliced into 4 μm sections. The sections were then baked at 60°C for 1 hour, dewaxed in xylene and dehydrated in gradient alcohol. Thereafter, the sections were incubated in 3% H_2_O_2_ at 37°C for 30 minutes to block endogenous peroxidase activity. Subsequently, the sections were blocked with 10% goat serum for 15 minutes and incubated overnight at 4°C with primary anti‐PDCD4 antibody (1:1000, Abcam) or primary anti‐OCN (osteocalcin) antibody (1:1000, Abcam), followed by incubation at 37°C for 1 hour with biotin‐labelled secondary antibodies (1:1000, Abcam). Subsequently, the sections were stained with diaminobenzidine for 10 minutes, counter‐stained with haematoxylin for 5 minutes, dehydrated using gradient alcohol, cleared in xylene, and mounted in neutral gum for assessment.

### 2.11 Statistical analysis

SPSS (IBM) was used to statistically analyse the data. All results were shown as mean ± standard deviation (SD). Student's *t* test was used to compare two groups, while one‐way ANOVA followed by Tukey's test as the post hoc test was used to compare multiple groups. A *P* value of <.05 was considered statistically significant.

## 3 RESULTS

### 3.1 Establishment of a rat model of ONFH and the characterization of hiPS‐MSC‐Exos

**Figure 1:**
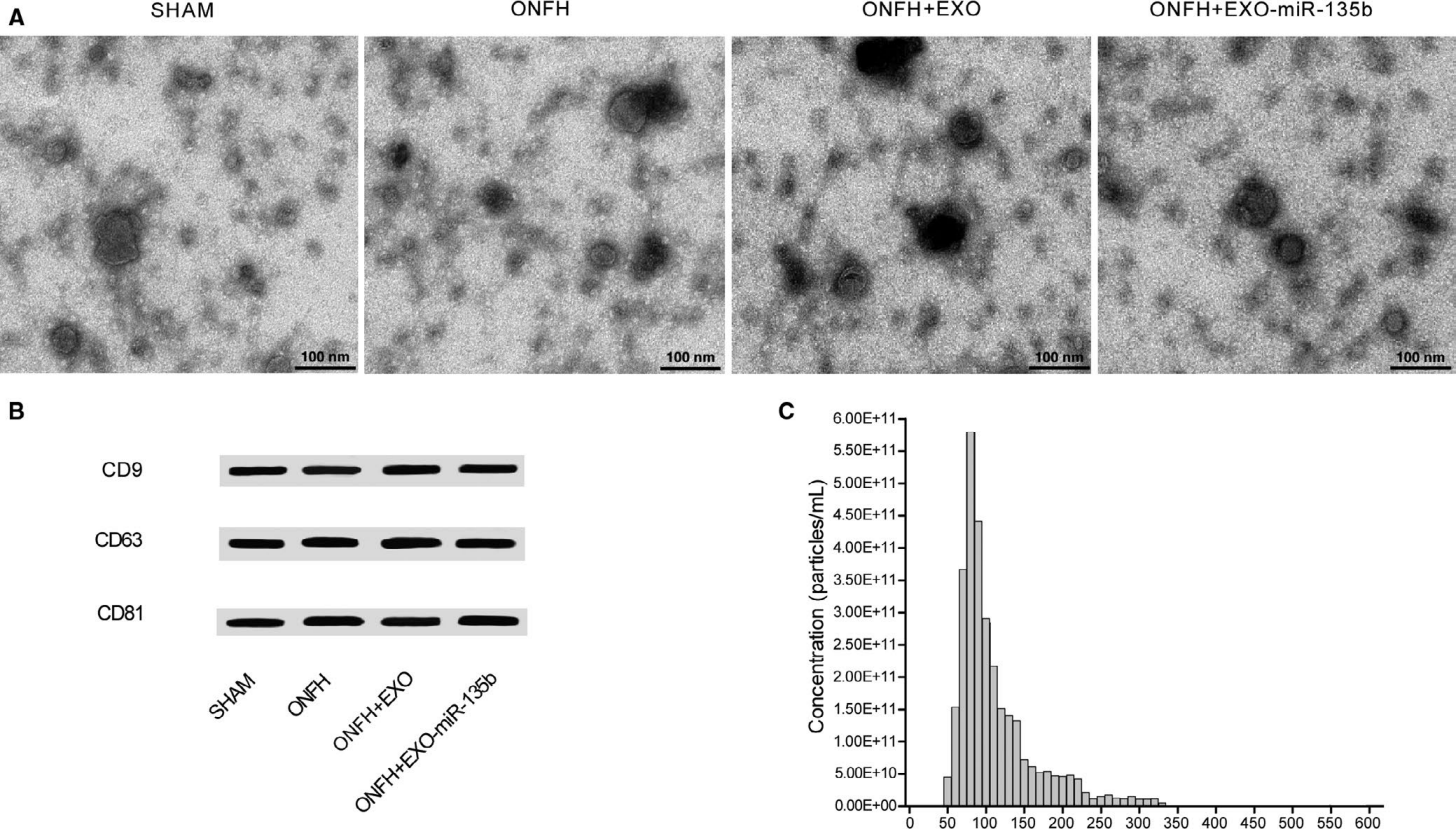
Characterization of hiPS‐MSC‐Exos. A, TEM showed hiPS‐MSC‐Exos with a spheroidal morphology and sizes ranging from 20 to 100 nm; B, Western blot analysis showed the expression of exosomal surface markers including CD9, CD63 and CD81; C, Nanoparticle analysis showed hiPS‐MSC‐Exos with sizes ranging from 20 to 100 nm and a mean concentration of 1 × 10^12^ particles/mL

During the characterization of hiPS‐MSC‐Exos, they displayed a spheroidal morphology with sizes ranging from 20 to 100 nm (Figure 1A). In a subsequent Western blot analysis to evaluate the expression of surface markers, including CD9, CD63 and CD81, on these exosomes, the abnormal enrichment of these proteins was observed (Figure 1B). Meanwhile, the results of nanoparticle analysis (Figure 1C) also showed the exosomes had a mean concentration of 1 × 10^12^ particles/mL and sizes ranging from 20 to 100 nm.

### 3.2 Effect of hiPS‐MSC‐Exos and miR‐135b on bone loss

**Figure 2:**
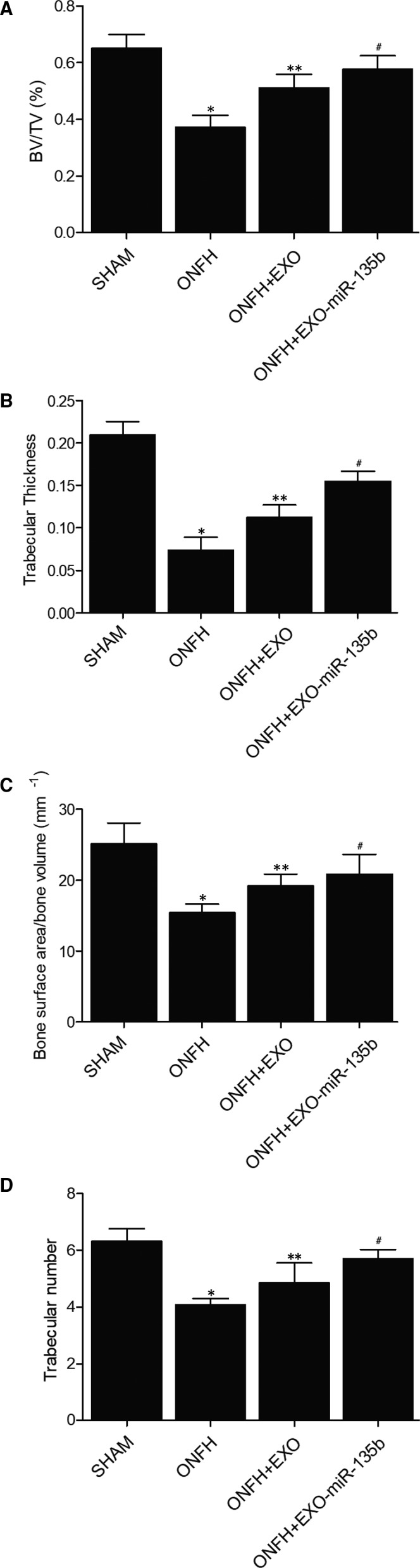
Effect of hiPS‐MSC‐Exos and miR‐135b on bone loss (*P value < .05 compared with the SHAM group; **P value < .05 compared with the ONFH group; ^#^ *P* value < .05 compared with the ONFH + EXO group). A, Quantification of BV/TV in the SHAM group, ONFH group, ONFH + EXO group and ONFH + EXO + miR‐135b group; B, Quantification of trabecular thickness in the SHAM group, ONFH group, ONFH + EXO group and ONFH + EXO + miR‐135b group; C, Quantification of bone surface area/bone volume in the SHAM group, ONFH group, ONFH + EXO group and ONFH + EXO + miR‐135b group; D, Number of trabecular in the SHAM group, ONFH group, ONFH + EXO group and ONFH + EXO + miR‐135b group

To evaluate the effect of hiPS‐MSC‐Exos and miR‐135b in the treatment of ONFH, trabecular bone structure of the femoral head was assessed by micro‐CT. As shown in Figure 2, the indicators related to bone structure, including bone volume/total volume (BV/TV) (Figure 2A), trabecular thickness (Figure 2B), bone surface area/bone volume (Figure 2C) and trabecular number (Figure 2D), were significantly inhibited in the ONFH group compared with those in the SHAM group, while the administration of exosomes in ONFH rats alleviated the severity of bone loss to a certain degree. In addition, the concomitant administration of miR‐135b and exosomes into ONFH rats reinforced the effect of exosomes and further alleviated the severity of bone loss. Therefore, hiPS‐MSC‐Exos and miR‐135b showed a synergistic and positive effect in the treatment of ONFH.

### 3.3 Differentiated expressions of miR‐135b, PDCD4 mRNA/protein and caspase‐3 in different animal groups

**Figure 3:**
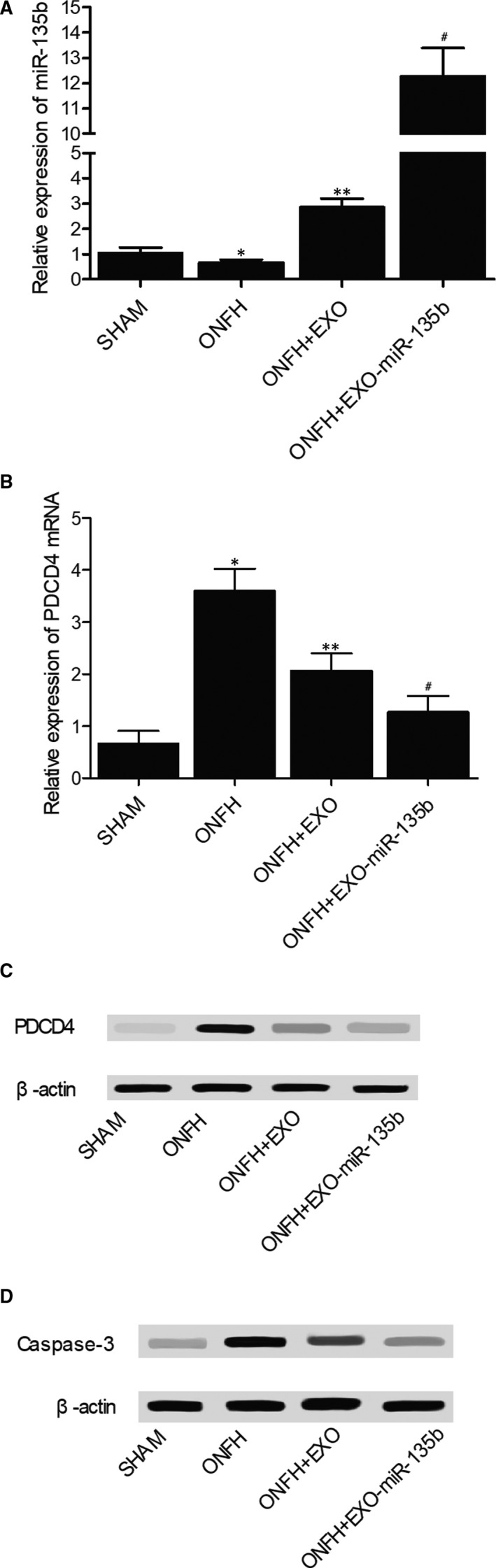
Expression of miR‐135b, PDCD4 mRNA/protein and caspase‐3 in the SHAM group, ONFH group, ONFH + EXO group and ONFH + EXO+miR‐135b group (*P value < .05 compared with the SHAM group; **P value < .05 compared with the ONFH group; ^#^P value < .05 compared with the ONFH + EXO group). A, Real‐time PCR results regarding the relative expression of miR‐135b in the SHAM group, ONFH group, ONFH + EXO group and ONFH + EXO + miR‐135b group; B, Real‐time PCR results regarding the relative expression of PDCD4 mRNA in the SHAM group, ONFH group, ONFH + EXO group and ONFH + EXO + miR‐135b group; C, Western blot results regarding the expression of PDCD4 protein in the SHAM group, ONFH group, ONFH + EXO group and ONFH + EXO + miR‐135b group; D, Western blot results regarding the expression of caspase‐3 in the SHAM group, ONFH group, ONFH + EXO group and ONFH + EXO + miR‐135b group

**Figure 4:**
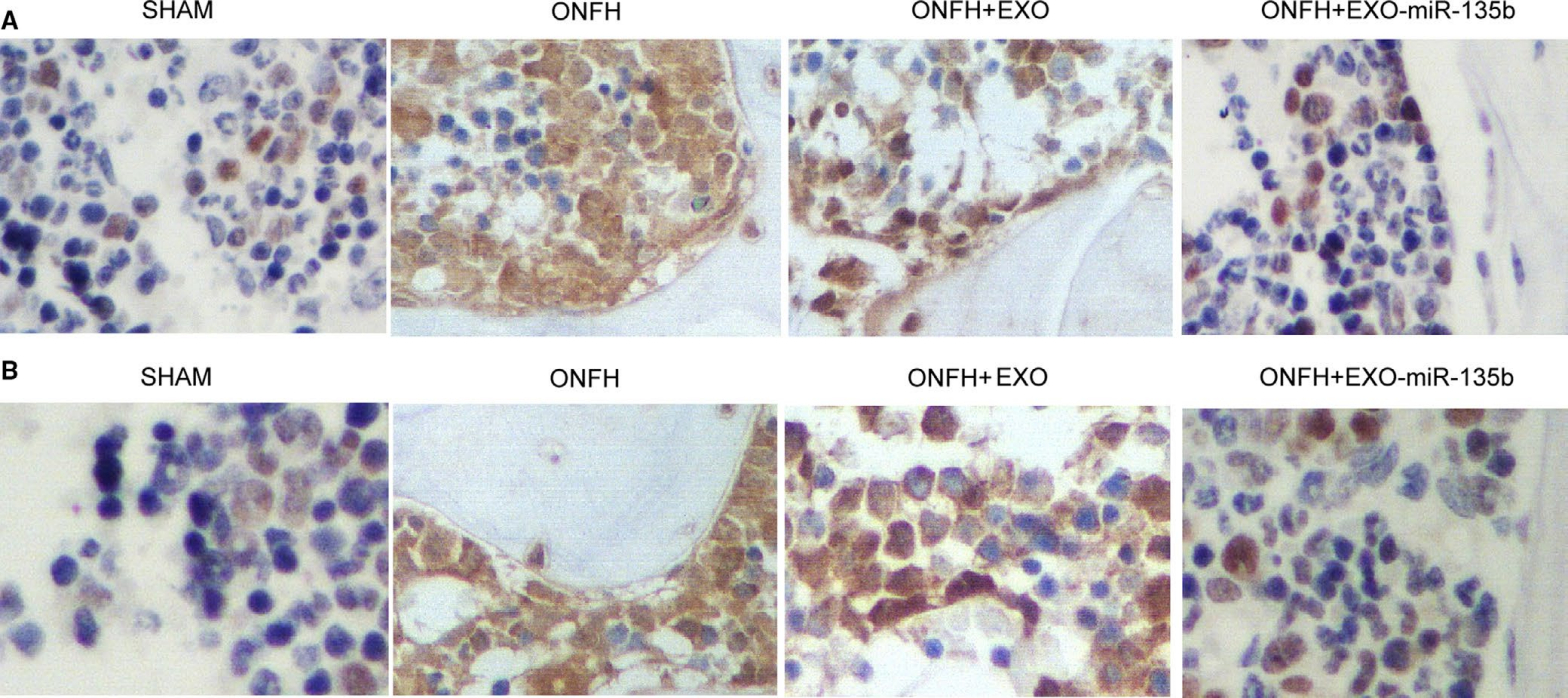
PDCD4 protein expression and osteocyte apoptosis in the animal groups. A, IHC results regarding the expression of PDCD4 protein in the SHAM group, ONFH group, ONFH + EXO group and ONFH + EXO + miR‐135b group. B, TUNEL assay results regarding the apoptosis index of osteocytes in the SHAM group, ONFH group, ONFH + EXO group and ONFH + EXO + miR‐135b group

The relative expression of miR‐135b and PDCD4 mRNA was determined via real‐time PCR. As shown in Figure 3A and compared with that in the SHAM group, the relative expression of miR‐135b was significantly up‐regulated in the ONFH + EXO group and evidently suppressed in the ONFH group, while the expression of miR‐135b was further increased in the ONFH + EXO + miR‐135b group. Interestingly, the expression of PDCD4 mRNA showed an opposite trend among these groups (Figure 3B). In addition, the administration of exosomes alone failed to completely alleviate the dysregulated expression of PDCD4 mRNA, while the concomitant administration of exosomes and miR‐135b fully inhibited the excessive expression of PDCD4 mRNA. Furthermore, in the Western blot analysis (Figure 3C) and IHC assay (Figure 4A), the ONFH group also presented a higher intensity of PDCD4 protein, while the concomitant treatment of exosomes and miR‐135b greatly reduced the intensity of PDCD4 protein. Additionally, the protein intensity of caspase‐3 (Figure 3D) showed the same trend as that of PDCD4 protein. Therefore, it can be concluded that the expression of miR‐135b, PDCD4 and caspase‐3 is affected during the treatment of ONFH by exosomes and miR‐135b.

### 3.4 In vivo effects of hiPS‐MSC‐Exos and miR‐135b on osteocyte apoptosis and OCN level

**Figure 5:**
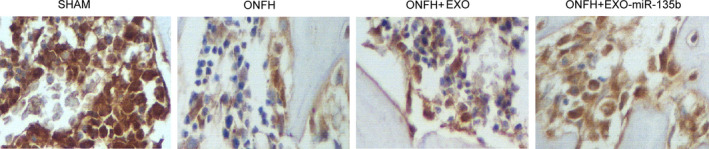
IHC results regarding the expression of OCN in the SHAM group, ONFH group, ONFH + EXO group and ONFH + EXO + miR‐135b group

In the TUNEL assay (Figure 4B), the apoptosis index of osteocytes was the highest in the ONFH group, while the administration of exosomes reduced the value of apoptosis index in ONFH rats to a certain degree. In addition, the co‐administration of exosomes and miR‐135b further reduced the value of apoptosis index in ONFH rats. Also, as shown in Figure 5, the IHC results of the expression of OCN in the rat groups also indicated that the concomitant treatment of exosomes and miR‐135b evidently promoted the suppressed level of OCN protein in ONFH rats. Therefore, the concomitant administration of exosomes and miR‐135b could most significantly alleviate the apoptosis of osteocytes while obstructing the down‐regulation of OCN in the treatment of ONFH.

### 3.5 In vitro effects of hiPS‐MSC‐Exos and miR‐135b on cell apoptosis and the expression of PDCD4/caspase‐3

**Figure 6:**
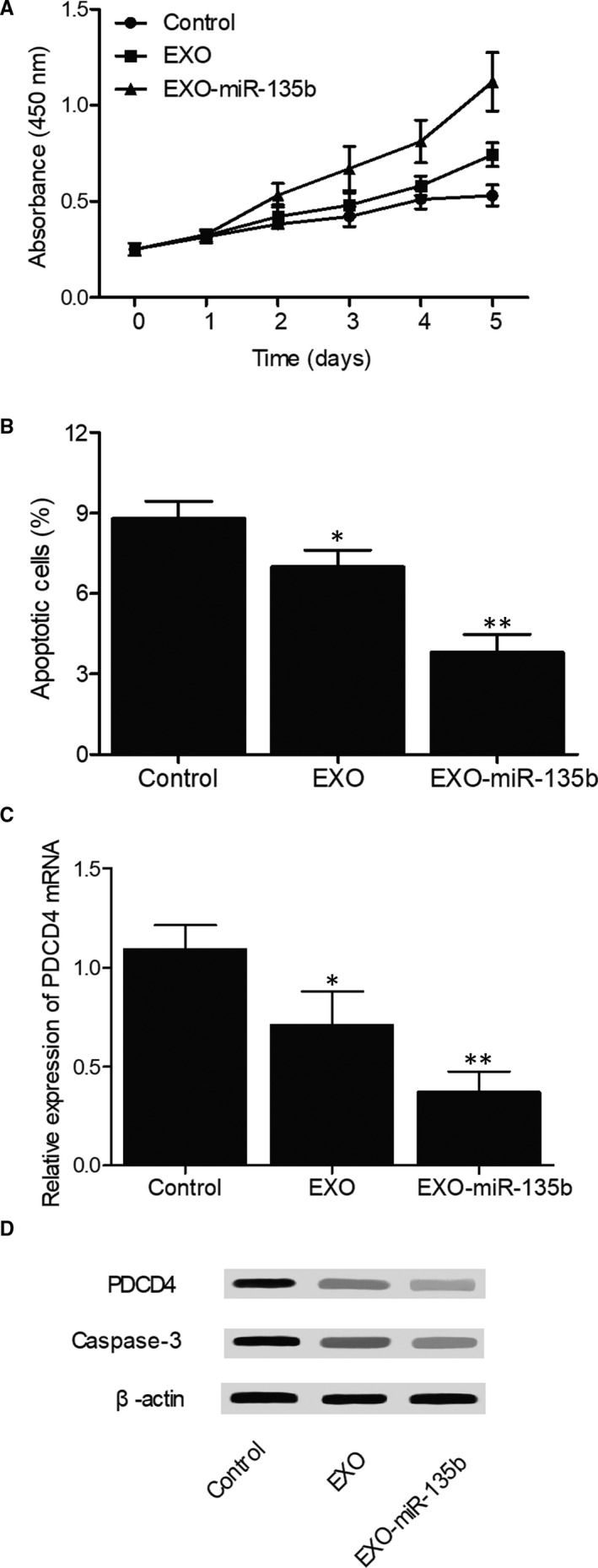
Effects of hiPS‐MSC‐Exos and miR‐135b on cell apoptosis and the expression of PDCD4/caspase‐3 in MG‐63 cells (*P value < .05 compared with the control group; **P value < .05 compared with the EXO group). A, MTT assay results regarding the proliferative capacity of MG‐63 cells treated with hiPS‐MSC‐Exos, hiPS‐MSC‐Exos + miR‐135b and the control; B, Flow cytometry results regarding the percentage of apoptotic cells in MG‐63 cells treated with hiPS‐MSC‐Exos, hiPS‐MSC‐Exos + miR‐135b and the control; C, Real‐time PCR results regarding the relative expression of PDCD4 mRNA in MG‐63 cells treated with hiPS‐MSC‐Exos, hiPS‐MSC‐Exos + miR‐135b and the control; D, Western blot results regarding the expression of PDCD4 protein and caspase‐3 in MG‐63 cells treated with hiPS‐MSC‐Exos, hiPS‐MSC‐Exos + miR‐135b and the control

**Figure 7:**
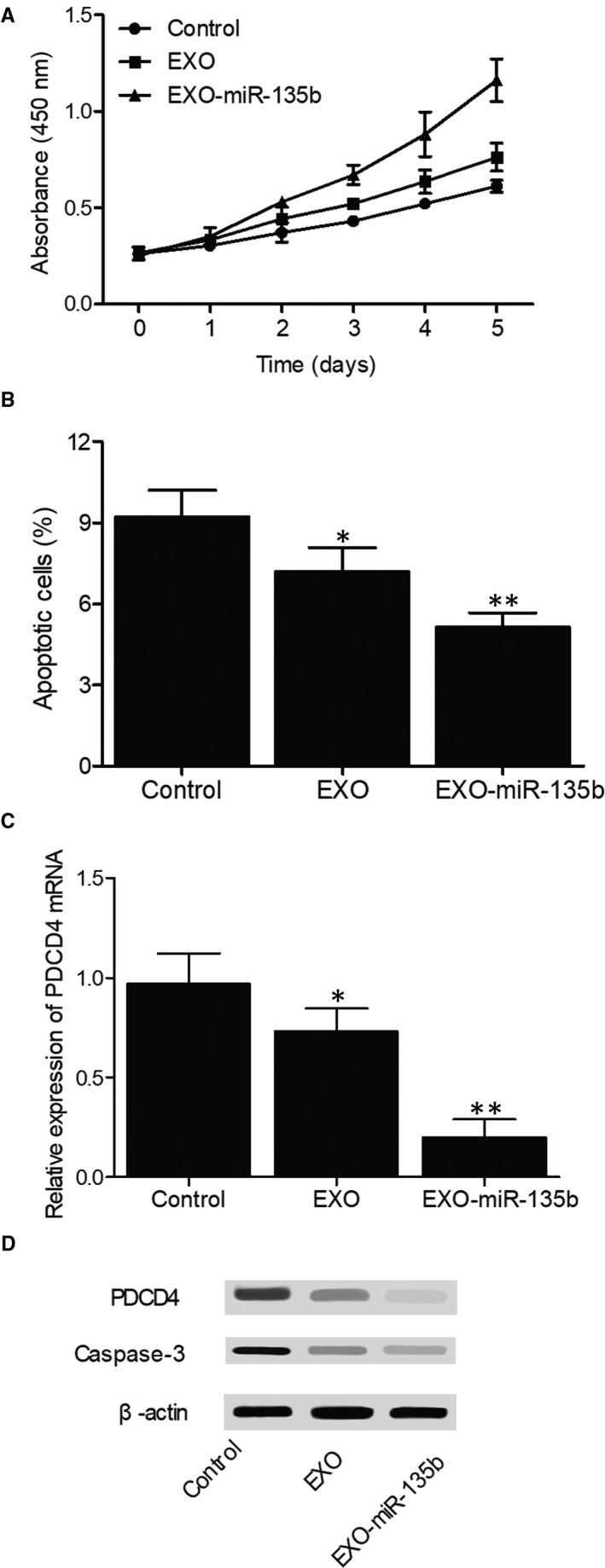
Effects of hiPS‐MSC‐Exos and miR‐135b on cell apoptosis and the expression of PDCD4/caspase‐3 in U‐2 cells (*P value < .05 compared with the control group; **P value < .05 compared with the EXO group). A, MTT assay results regarding the proliferative capacity of U‐2 cells treated with hiPS‐MSC‐Exos, hiPS‐MSC‐Exos + miR‐135b and the control; B, Flow cytometry results regarding the percentage of apoptotic cells in U‐2 cells treated with hiPS‐MSC‐Exos, hiPS‐MSC‐Exos + miR‐135b and the control; C, Real‐time PCR results regarding the relative expression of PDCD4 mRNA in U‐2 cells treated with hiPS‐MSC‐Exos, hiPS‐MSC‐Exos + miR‐135b and the control; D, Western blot results regarding the expression of PDCD4 protein and caspase‐3 in U‐2 cells treated with hiPS‐MSC‐Exos, hiPS‐MSC‐Exos + miR‐135b and the control

In the MTT assay, the proliferation of MG‐63 cells treated with hiPS‐MSC‐Exos or hiPS‐MSC‐Exos + miR‐135b (Figure 6A) showed a dose‐dependent increase at all time points. In addition, the presence of miR‐135b reinforced the effect of hiPS‐MSC‐Exos. The results of flow cytometry showed that the percentage of apoptotic MG‐63 cells (Figure 6B) in the EXO group was reduced compared with that in the control group, while the percentage of apoptotic MG‐63 cells in the EXO + miR‐135b group was the lowest. The flow cytometry data plots were presented in Supplementary Figure 1. Moreover, in the presence of hiPS‐MSC‐Exos, the relative expression of PDCD4 mRNA (Figure 6C) was down‐regulated, and the co‐administration of miR‐135b further inhibited the mRNA expression of PDCD4. The results of Western blot analysis also showed that the protein expression of caspase‐3 and PDCD4 (Figure 6D) was evidently reduced in the presence of hiPS‐MSC‐Exos and miR‐135b. Similar results were also observed in U‐2 cells (Figure 7), indicating that the therapeutic effect of exosomes and miR‐135b in the treatment of ONFH may be associated with their ability to regulate the expression of PDCD4 and caspase‐3.

### 3.6 The establishment of a miR‐135b/PDCD4 molecular pathway

**Figure 8:**
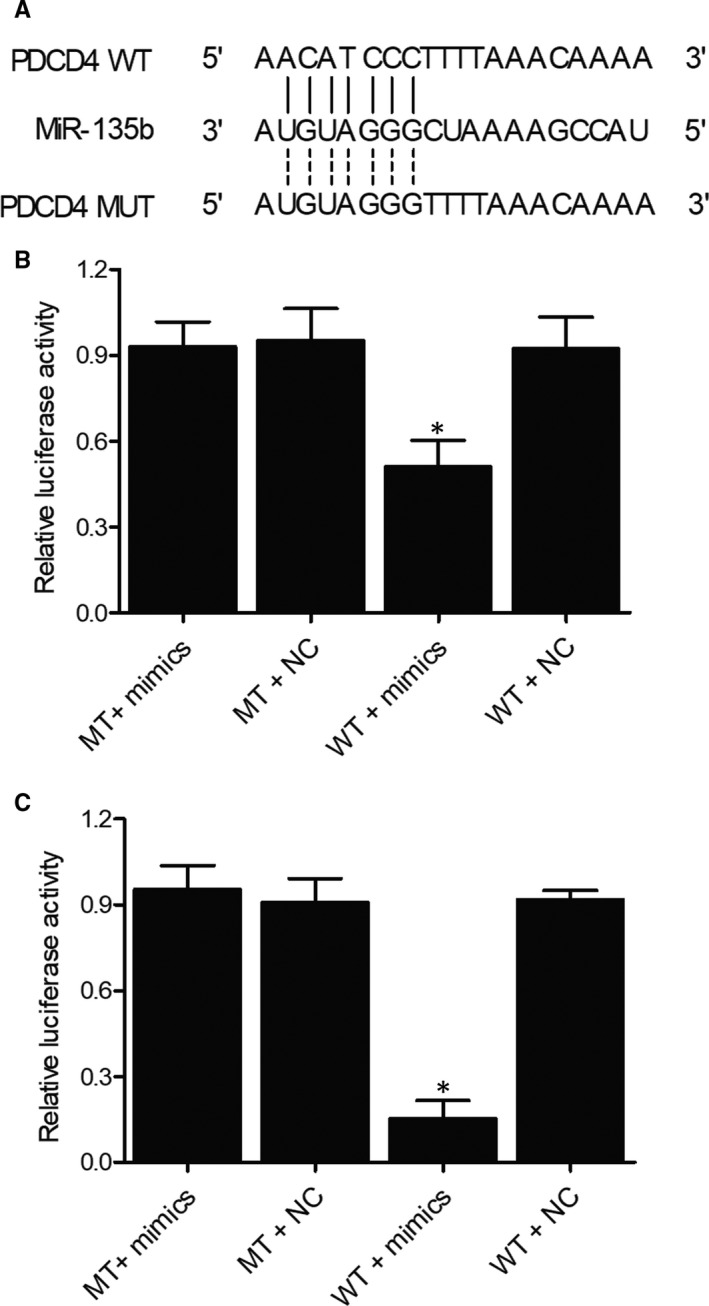
The establishment of a miR‐135b/PDCD4 molecular pathway (*P value < .05 compared with the MT + mimics group). A, Computational analysis of the sequences of miR‐135b and PDCD4 mRNA; B, Luciferase activity of MG‐63 cells transfected with wild‐type PDCD4 mRNA + miR‐135b, mutant PDCD4 mRNA + miR‐135b, wild‐type PDCD4 mRNA + control miRNA, and mutant PDCD4 mRNA + control miRNA; C, Luciferase activity of U‐2 cells transfected with wild‐type PDCD4 mRNA + miR‐135b, mutant PDCD4 mRNA + miR‐135b, wild‐type PDCD4 mRNA + control miRNA, and mutant PDCD4 mRNA + control miRNA

Computational analysis was carried out to identified a ‘seed sequence’ of miR‐135b on the 3′UTR of PDCD4 mRNA (Figure 8A). In addition, the results of luciferase assay only showed reduced luciferase activity in MG‐63 (Figure 8B) and U‐2 (Figure 8C) cells co‐transfected with wild‐type PDCD4 mRNA and miR‐135b. Therefore, it can be concluded that PDCD4 mRNA is targeted by miR‐135b and the reinforcing effect of miR‐135b in the administration of hiPS‐MSC‐Exos is mediated by its inhibition on PDCD4expression.

## 4 DISCUSSION

In this study, a rat model of ONFH was established and treated with MSC‐Exos or EXO‐miR‐135b. It was found that bone indicators, including BV/TV, trabecular thickness, bone surface area/bone volume and trabecular number, were significantly reduced in the ONFH group compared with those in the SHAM group, while the values of these indicators in ONFH + EXO and ONFH + EXO + miR‐135b groups were increased to a certain degree, indicating a positive effect of hiPS‐MSC‐Exos and miR‐135b in the treatment of ONFH.

Yan et al^20^ showed that the delivery of MSCs into the femoral head can accelerate bone repair in an animal model of ONFH. Tabatabaee et al^21^ showed that the delivery of autologous BMSCs into patients with early‐stage ONFH can improve their prognosis. As small EVs, exosomes are derived from late endosomes as MVBs.^22^ At first, exosomes were thought as containers used to ship out unwanted membranous proteins or intracellular components from reticulocytes.^23^ Recently, exosomes have been shown to play important roles in the development of autoimmune diseases and cancer. Therefore, exosomes are now considered as a novel therapeutic tool that can be used in the treatment of neurological disorders including Parkinson's disease.^24^ In particular, exosomes originated from several types of cells are shown to act as carriers of signalling transduction by transporting cell‐specific miRNAs, DNAs, RNAs, lipids and proteins.^25^ In this study, we showed that osteocytes became highly apoptotic in the ONFH group, while the presence of hiPS‐MSC‐Exos and miR‐135b alleviated the apoptosis of osteocytes. In addition, the treatment of osteocytes spontaneously with hiPS‐MSC‐Exos and miR‐135b most significantly reduced the value of apoptosis index. Meanwhile, the administration of hiPS‐MSC‐Exos and miR‐135b increased the expression of miR‐135b and reduced the level of PDCD4 mRNA/protein. In addition, the rats in the ONFH + EXO and ONFH + EXO + miR‐135b groups presented reduced expression of caspase‐3 protein. Therefore, hiPS‐MSC‐Exos and miR‐135b treatment can affect the expression of miR‐135b, PDCD4 and caspase‐3 during the treatment of ONFH.

Guo et al have shown that the preventive treatment of rats using exosomes released from human synovial‐derived mesenchymal stem cells (SMSC‐Exos) hindered the development of glucocorticoid‐induced ONFH. After their release, SMSC‐Exos are internalized by BMSCs and enhance the proliferation of BMSCs while inhibiting their apoptosis.^19^ By transfecting BMSCs with mutant HIF‐1α, Li et al showed that HIF‐1α is essential for bone development. Furthermore, exosomes released from transfected BMSCs can enhance bone repair in steroid‐induced ONFH.^26^

Increased expression of miR‐135b is observed in many cancers.^27^, ^28^, ^29^ Real‐time PCR and microarray analysis both showed that the expression of miR‐135b is significantly up‐regulated in non‐small cell lung cancer and head and neck squamous cell carcinoma.^27^, ^28^ Studies also demonstrated that the expression of miR‐135b is significantly up‐regulated in both carcinomas and adenomas.^29^ Moreover, the expression of miR‐135b is up‐regulated during the malignant transformation of normal tissues, indicating that the deregulation of miR‐135b is involved in the onset of CRC. Located on 1q32.1, miR‐135b is encoded by the LEMD1 gene. Interestingly, the DNA copy number of 1q32.1 usually increases during the progression of CRC, while the expression of LEMD1 is highly increased in CRC tissues.^30^, ^31^ Furthermore, past studies indicated that miR‐135b can promote the malignant transformation of colorectal tissues and is required for the proliferation of placental cells.^32^ It was also reported that miR‐135b is implicated in the progression of many cancers.^33^ In particular, overexpression of miR‐135b is observed in lung, breast, and colon cancers.^33^ It was also shown that, as a tumour inhibitor involved in the regulation of cell invasion, cell migration, and c‐Myc expression, miR‐135b is apparently down‐regulated in osteosarcoma.^34^ It has also been shown that the silence of miR‐135b expression induces the apoptosis of CRC cells, although the relationship between miR‐135b expression and the development of chemo‐resistance in CRC cells remains unknown.^35^ It is suspected that miR‐135b can reduce the chemosensitivity and inhibit the apoptosis of colorectal cancer cells in vivo.^33^ In this study, the proliferation of cells treated with hiPS‐MSC‐Exos showed a dose‐dependent increase, while the treatment with miR‐135b further reinforced the effect of hiPS‐MSC‐Exos. Moreover, the presence of hiPS‐MSC‐Exos and miR‐135b reduced the percentage of apoptotic cells compared with that in the control group. Additionally, the cells treated with hiPS‐MSC‐Exos showed a reduced level of PDCD4 mRNA/protein, while the co‐administration of miR‐135b and hiPS‐MSC‐Exos further inhibited the expression of PDCD4 mRNA/protein.

The up‐regulation of PDCD4 was discovered in cells undergoing apoptosis, and PDCD4 has been shown to act as a novel tumour inhibitor and its down‐regulation is observed in many cancers.^36^, ^37^ Furthermore, as a gene ubiquitously expressed in normal tissues, PDCD4 is up‐regulated in both healthy and apoptotic cells.^37^ In addition, the overexpression or silencing of PDCD4 can greatly impact cell survival and growth.^38^ Although the mechanisms underlying the effect of PDCD4 remain unknown, it is believed that the PDCD4 protein in the cytosol can interact with eukaryotic translation initiation factor 4A (eIF4A) through its MA‐3 domain and suppress the activity of its helicase, thus suppressing cap‐dependent translation.^39^ In addition, PDCD4 controls gene transcription by suppressing the activity of activator protein‐1 (AP‐1) in the nucleus.

## 5 CONCLUSION

In summary, we demonstrated here for the first time that miR‐135b derived from exosomes secreted from MSCs alleviated the severity of ONFH in rats by reducing the level of PDCD4‐induced apoptosis of osteoblasts. We also showed that, by directly targeting the 3’UTR of PDCD4, miR‐135b derived from exosomes secreted from MSCs suppressed PDCD4 expression and hence played an important role in the treatment of ONFH.

## CONFLICT OF INTEREST

None.

## AUTHOR CONTRIBUTION

**Xiang Zhang:** Conceptualization (equal); Investigation (equal); Methodology (equal); Supervision (equal); Visualization (equal); Writing‐review & editing (equal). **Jiong‐ming You:** Investigation (equal); Methodology (equal); Software (equal); Visualization (equal). **Xiao‐jun Dong:** Investigation (equal); Resources (equal); Visualization (equal). **Yang Wu:** Conceptualization (equal); Funding acquisition (equal); Methodology (equal); Project administration (equal); Supervision (equal); Writing‐original draft (equal).

## Supporting information

Figure S1

## Data Availability

The data sets generated during and/or analysed during the current study are available from the corresponding author on reasonable request.
