# Supplementary figures and images for: Administration of mircoRNA‐135b‐reinforced exosomes derived from MSCs ameliorates glucocorticoid‐induced osteonecrosis of femoral head (ONFH) in rats
Source: J Cell Mol Med. 2020 Oct 22;24(23):13973–83. doi: 10.1111/jcmm.16006 (PMC7754047; doi:10.1111/jcmm.16006)

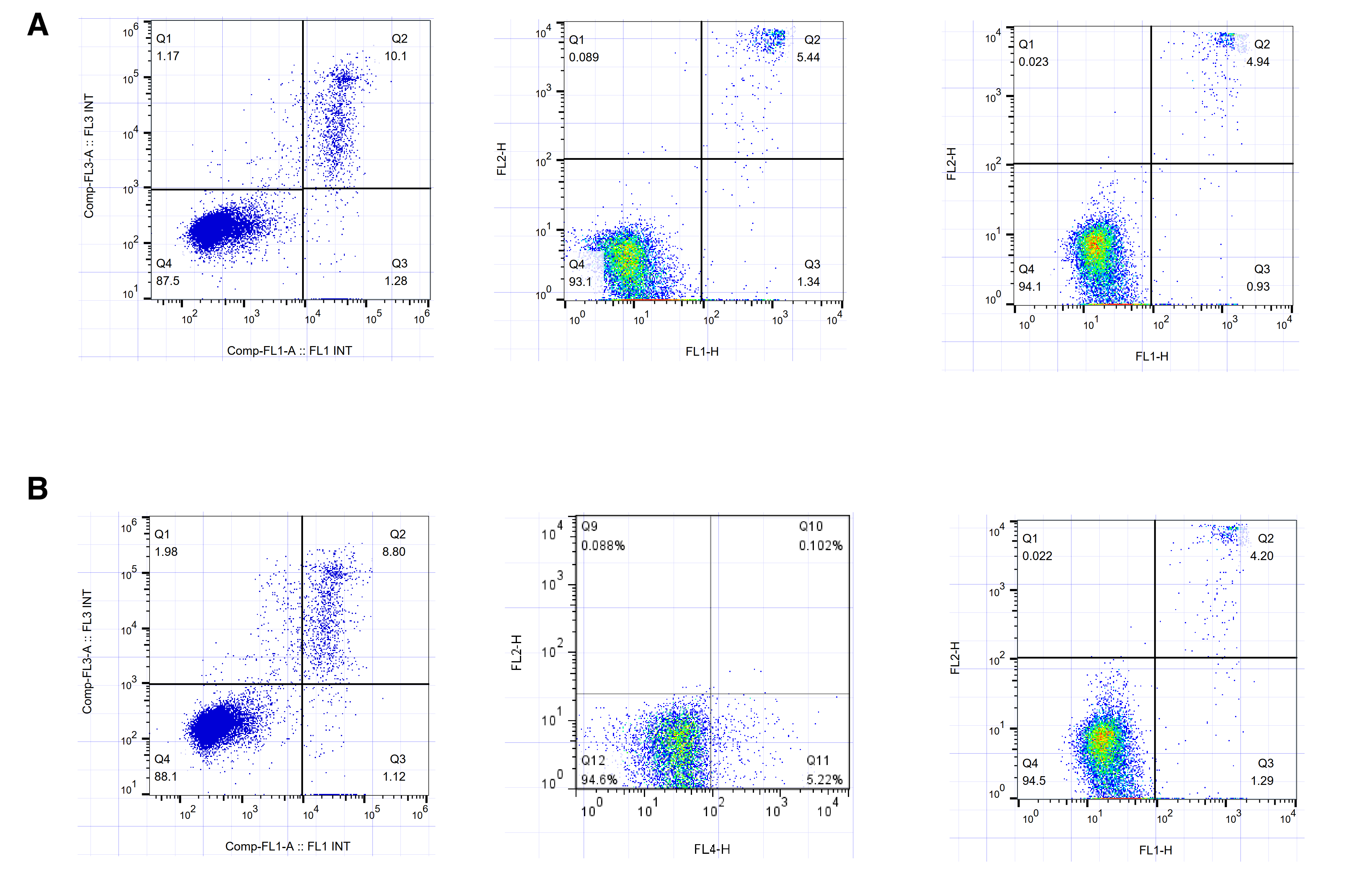

Supplement: Supplementary file 1 — Figure S1 [file JCMM-24-13973-s001.tif]
